# Polymorphisms in the *mTOR* Gene and Risk of Sporadic Prostate Cancer in an Eastern Chinese Population

**DOI:** 10.1371/journal.pone.0071968

**Published:** 2013-08-05

**Authors:** Qiaoxin Li, Chengyuan Gu, Yao Zhu, Mengyun Wang, Yajun Yang, Jiucun Wang, Li Jin, Mei-Ling Zhu, Ting-Yan Shi, Jing He, Xiaoyan Zhou, Ding-wei Ye, Qingyi Wei

**Affiliations:** 1 Cancer Institute, Fudan University Shanghai Cancer Institute, Shanghai, China; 2 Department of Oncology, Shanghai Medical College, Fudan University, Shanghai, China; 3 Department of Urology, Fudan University Shanghai Cancer Institute, Shanghai, China; 4 Ministry of Education Key Laboratory of Contemporary Anthropology, State Key Laboratory of Genetic Engineering, School of life Sciences, Fudan University, Shanghai, China; 5 Fudan-Taizhou Institute of Health Sciences, Taizhou, Jiangsu, China; 6 Department of Pathology, Shanghai Medical College, Fudan University, Shanghai, China; 7 Department of Epidemiology, The University of Texas MD Anderson Cancer Center, Houston, Texas, The United States of America; MOE Key Laboratory of Environment and Health, School of Public Health, Tongji Medical College, Huazhong University of Science and Technology, China

## Abstract

**Background:**

The *mTOR* gene regulates cell growth by controlling mRNA translation, ribosome biogenesis, autophagy, and metabolism. Abnormally increased expression of *mTOR* was associated with carcinogenesis, and its functional single nucleotide polymorphisms (SNPs) may regulate the expression of *mTOR* and thus contribute to cancer risk.

**Methodology/Principal Findings:**

In a hospital-based case-control study of 1004 prostate cancer (PCa) cases and 1051 cancer-free controls, we genotyped six potentially functional SNPs of *mTOR* (rs2536 T>C, rs1883965 G>A, rs1034528 G>C, rs17036508 T>C, rs3806317 A>G, and rs2295080 T>G) and assessed their associations with risk of PCa by using logistic regression analysis.

**Conclusions/Significances:**

In the single-locus analysis, we found a significantly increased risk of PCa associated with *mTOR* rs2536 CT/CC and rs1034528 CG/CC genotypes [adjusted OR = 1.42 (1.13–1.78), *P* = 0.003 and 1.29 (1.07–1.55), *P* = 0.007), respectively], compared with their common homozygous genotypes, whereas *mTOR* rs2295080 GT/GG genotypes were associated with a decreased risk of PCa [adjusted OR = 0.76 (0.64–0.92), *P* = 0.003], compared with wild-type TT genotypes. In the combined analysis of the six SNPs, we found that individuals carrying two or more adverse genotypes had an increased risk of PCa [adjusted OR = 1.24 (1.04–1.47), *P* = 0.016], compared with individuals carrying less than two adverse genotypes. In the multiple dimension reduction analysis, body mass index (BMI) was the best one-factor model with the highest CVC (100%) and the lowest prediction error (42.7%) among all seven factors. The model including an interaction among BMI, rs17036508, and rs2536 was the best three-factor model with the highest CVC (100%) and the lowest prediction error of 41.9%. These findings suggested that *mTOR* SNPs may contribute to the risk of PCa in Eastern Chinese men, but the effect was weak and needs further validation by larger population-based studies.

## Introduction

Prostate cancer (PCa) is the second most frequently diagnosed cancer and the sixth leading cause of cancer death in males according to the latest report released by the International Agency for Research on Cancer (IARC) in 2008 [1]. It has been well established that PCa is one of the pronounced geographically and ethnically related human malignancies, with a much higher incidence observed in the Western world than in Asian countries [2]. Recently, accumulated evidence from genome-wide association studies (GWASs) suggests that more than 40 single nucleotide polymorphisms (SNPs) are associated with human PCa risk, some of which were also confirmed in Chinese male populations. However, almost all the candidate SNPs are reported to be in weak associations with PCa risk to date [3]–[6]. Therefore, it is still not fully understood to what extent genetic factors and their interactions with environmental attributes may play a role in the etiology of PCa.

The phosphoinositide-3 kinase-AKT-mammalian target of rapamycin pathway (PI3K/AKT/mTOR) is a major pathway controlling cell growth and tumogenesis [7], [8]. As a key downstream effector of PI3K/AKT/mTOR pathway, the mTOR has been confirmed to be a central regulator of vital cellular processes, such as cell growth, proliferation, metabolism, migration, and apoptosis, based on the *in vivo* and *in vitro* investigations [9]–[12]. Structurally, mTOR contains several important domains across the whole protein, of these, the rapamycin-binding domain and the kinase domain was considered closely relevant to carcinogenesis [13]. Additionally, several studies have demonstrated that mTOR targeted therapies can be designed to block the induction of the proliferative, prosurvival, and oncogenic functions of *mTOR* [14]. Therefore, it was speculated that *mTOR* is a possible driver gene in carcinogenesis, and a promising target point and prognosis marker in cancer treatment as well.

Somatic aberrations of PI3K/AKT/mTOR pathway genes have been commonly observed in a variety of malignancies, including PCa [8]. And the mutations in the *mTOR* gene have been identified in a few human cancers [15]; however, the mechanism has not been well established to date. PCa harboring almost the same well-known mutations often presents with heterogeneous clinicpathologic characteristics. By the same token, genetic factors, such as naturally occurring polymorphic genetic variants or SNPs in *mTOR*, may be contributing to the variation in individual susceptibility to PCa and the progression of this disease.

Given that mTOR is one of the most important downstream components of the mTOR pathway, which can also receive signals from other pivotal pathways. Several studies have demonstrated that mTOR can serve as a promising therapeutic target in the future cancer treatment. And there have been few studies to date addressing the role of common, functional variants in the mTOR gene as PCa susceptibility factors, together with some variations of other pivotal genes in this pathway have been investigated as weak or null associations with cancer risk. we performed a case-control study by genotyping six potential functional SNPs in *mTOR* using genomic DNA from 1004 patients with prostate adenocarcinoma and 1051 cancer-free controls in an Eastern Chinese Han population. We tested the hypothesis that risk of PCa may be associated with SNPs in the *mTOR* gene and their interactions with environmental factors.

## Materials and Methods

### Patients and controls

We recruited PCa patients and the matched cancer-free controls from genetically unrelated Chinese Han participants between January 2008 and January 2012. This analysis included 1004 patients who were inhabitants of the administrative regions of eastern China (including Shanghai city, Zhejiang province, Jiangsu province and the surrounding areas) and have been histopathologically confirmed primary prostate adenocarcinoma at Fudan University Shanghai Cancer Center (FUSCC). All cases had received no prior chemotherapy or radiotherapy upon recruitment. The clinical stages were determined and categorized into stage I (T1a-bN0M0), stage II (T1c-2N0M0), stage III (T3-4N0M0), and stage IV (T1c-4N1M0-1 or T1-4N0-1M1) according to the Tumor-Node-Metastasis system, and pathological grades of the PCa were determined according to the WHO criteria [16]. The entire document, including the Gleason score, serum PSA level at diagnosis, and clinical staging (TNM) was abstracted from the archival medical records. The male control group was comprised of 1051 cancer-free individuals, frequency-matched with the cases by age (±5 years) and geographical regions, recruited from the Taizhou longitudinal study (TZL) [17] during the same period. Individuals with a known test of serum PSA >4 ng/mL present with or without abnormal digital rectal examination were excluded from the control group.

All of the participants were interviewed with a self-administered questionnaire after a written informed consent was obtained. Blood samples were collected and processed, with a written informed consent from participants, as a routine practice by the Institutional Tissue Bank at Shanghai Cancer Institute (for cases) and the TZL study (for controls). Response rate was 92% and 91% for cases and controls, respectively. The research was approved by the Institutional Review Board of FUSCC.

### Single nucleotide polymorphisms selection

Among all of the reported *mTOR* SNPs, potentially functional SNPs of interest were selected from the NCBI dbSNP database (http://www.ncbi.nlm.nih.gov/projects/SNP) and SNPinfo (http://snpinfo.niehs.nih.gov/snpfunc.htm) according to the following criteria: 1) the minor allele frequency (MAF) reported in HapMap was≥5% for Chinese populations; 2) affecting the functional regions of the gene, including transcription factor binding site (TFBS), potential miRNA binding site, splicing regulation locus, and stop codon; 3) the linkage disequilibrium (LD) coefficient *r* ^2^ <0.8 between SNPs; and) not included in the published GWASs studies. Ultimately, six variants were selected for the present study, including rs2536 T>C, rs1883965 G>A, rs1034528 G>C, rs17036508 T>C, rs3806317 A>G, and rs2295080 T>G, of which four (rs1034528 G>C, rs1883965 G>A, rs2295080 T>G, and rs3806317 A>G) located in the first intron region may affect the transcription factor binding site (TFBS) activity, two (rs2536 T>C and rs17036508 T>C) located in the 3′-untranslated region (3′ UTR) region may affect the miRNA binding site activity, and SNP rs17036508 T>C locus also predicted locate at the potential splicing site. Bioinformatics analysis was performed with HaploView software 4.2 to estimate the haplotype block for Chinese population (CHB) data of HapMap (HapMap Data Rel 27 Phase II+III), and no LD was found between any of these SNPs described above. All these six selected SNPs were genotyped by the TaqMan real-time PCR method as described previously [17], and the results with >98% call rates and 100% concordance for duplicated specimens were acceptable for further genotyping data analysis.

### Multifactor Dimensionality Reduction (MDR) Analysis

Evidence indicated that gene–gene and gene–environment interactions are difficult to be fully characterized by using logistic regression model. And statistic power would decrease and type II errors would increase when detecting interactions by LR in case-control studies with relatively small sample sizes [18]. By contrast, The MDR analysis can overcomes some of the limitations of logistic regression model for the interactions by collapsing high-dimensional data into a single dimensional variable with two levels. In the present study, we performed the MDR analysis, as described previously [19]. We used a model of 100-fold cross-validation and repeated the complete analysis for 10 times under different random seeds, and then the test was repeated 1000 times under the null hypothesis of no association. As a result, the model employing the minimized prediction error together with the maximized cross-validation consistency (CVC) was recommended. This analysis was performed by using the MDR V2.0 beta 8.2 software (http://www.multifactordimensionalityreduction.org/).

### Statistical analysis

Hardy-Weinberg equilibrium (HWE) for evaluation of genotype distributions of the controls was performed by a goodness-of fit χ^2^ test. Differences in the frequency distributions of the alleles, genotypes and the selected categorical variables between cases and controls were evaluated by Pearson's χ^2^ test under various genetic models (including dominant model, recessive model, and additive model). Crude and adjusted odds ratios (ORs) and 95% confidence intervals (CIs) were calculated according to the significant genetic models by univariate and multivariate unconditional logistic regression models, respectively, to evaluate associations between the genotypes and risk of Pca with and without adjustment for by confounding factors. Given the present study was single ethnicity, and all of the SNPs loci were agree with HEW, the confounding factors which should be adjusted for was age, smoking status, and body mass index (BMI). Further stratification analyses were conducted to calculate the associations of SNP genotypes with PCa risk by demographic and clinic-pathologic variables, followed by the homogeneity Q-tests to detect any difference in the risk estimates between the strata. Based on the observed genotypes, haplotype frequencies and individual haplotypes were generated using Statistical Analysis Software PROC HAPLOTYPE, with a reference group of common haplotype, to calculate ORs for haplotypes associated with PCa risk in logistic regression analysis. For all the significant findings observed in our study, we calculated the false-positive report probability (FPRP) with prior probabilities of 0.0001, 0.001, 0.01, 0.1 and 0.25 to detect the possible false-positive associations [20]. Statistical power was estimated to detect an OR of 1.50/0.67 (for a risk/protective effect), with an α level equal to the observed *P* value. Only significant results with FPRP value less than 0.2 were considered a noteworthy association. All statistical analyses were performed with SAS 9.1 statistical software (SAS, Cary, NC, USA). All *P* values were two-sided with a significance level of *P* <0.05.

## Results

### Characteristics of the subjects

The distributions of demographic characteristics of the subjects are presented in **Table 1**. Briefly, there were no statistical differences in the distributions of age and smoking status between 1004 cases and 1051 controls. The body mass index (BMI) for overweight (> 24.0 kg/m^2^) was more evident in controls than in cases (*P* < 0.0001), which was further adjusted for in subsequent multivariate logistic regression analyses. Among the case subjects, 178 (17.7%) cases were PSA≤10 ng/ml, 312 (31.1%) cases were Gleason score≤7 (3+4), and 601 (59.9%) cases were Gleason score≥7 (4+3). For tumor staging, five (0.5%) cases had stage I disease, 431 (42.9%) had stage II disease, 140 (13.9%) had stage III disease, and 351 (35.0%) had stage IV disease. However, some cases had missing data because of the insufficient documented records, including 87 (8.7%) lacking serum PSA values, 91 (9.1%) lacking Gleason scores, and 77 (7.7%) lacking clinical staging status.

**Table 1:** Distribution of demographic and clinical-pathologic characteristics of prostate cancer patients and cancer-free controls from Eastern Chinese men.

Variables	Cases No. (%)	Controls No. (%)	*P* a
All subjects	1004 (100)	1051 (100)	
Age, yr (Mean±SD)	69.0±8.16	69.0±8.96	0.141
≤69	510 (50.8)	494 (49.2)	
>69	568 (54.0)	483 (46.0)	
BMI (kg/m^2^)			< 0.0001
≤24	754 (75.1)	250 (24.9)	
>24	637 (60.6)	414 (39.4)	
Smoking status			0.572
Never	402 (40.0)	602 (60.0)	
Ever	408 (38.8)	643 (61.2)	
PSA value (ng/ml)			
≤10	178 (17.7)		
10–20	193 (19.2)		
>20	546 (54.4)		
Missing	87 (8.7)		
Gleason score			
≤7(3+4)	312 (31.1)		
≥7(4+3)	601 (59.9)		
Missing	91 (9.1)		
Stage of disease			
I	5 (0.5)		
II	431 (42.9)		
III	140 (13.9)		
IV	351 (35.0)		
Missing	77 (7.7)		

SD, standard deviation. BMI, body mass index.

a Two-sided chi-square tests were used to calculate differences in the frequency distribution of genotypes between cases and controls.

The results were in bold, if *P*<0.05.

### The *mTOR* allele and genotype distributions and associations with PCa risk

The genotype and allele distributions of the six selected SNPs among cases and controls are summarized in **Table 2**. The observed genotype frequencies of the six SNPs in controls agreed with the Hardy-Weinberg equilibrium. Furthermore, significant differences in genotype distributions were observed between cases and controls for rs2536 T>C (*P* = 0.007), rs1034528 G>C (*P* = 0.022), and rs2295080 T>G (*P* = 0.012). Interestingly, the heterozygote genotypes of the above three SNPs were more likely to be significantly associated with PCa risk with adjusted OR (95% CI) and *P* value of 1.45 (1.15–1.84) and 0.002 for rs2536 TC, 1.31 (1.08–1.59) and 0.005 for rs1034528 GC, and 0.77 (0.64–0.93) and 0.006 for rs2295080 TG, respectively, compared with their respective wild-type genotypes, respectively. Additionally, we also found significant associations with PCa risk for SNPs in special genetic models, including rs2536 T>C [additive: adjusted OR = 1.34 (1.08–1.66), *P* = 0.008; dominant: adjusted OR = 1.42 (1.13–1.78), *P* = 0.003]; rs1034528 G>C [additive: adjusted OR = 1.21 (1.03–1.42), *P* = 0.019; dominant: adjusted OR = 1.29 (1.07–1.55), *P* = 0.007]; and rs2295080 T>G [additive: adjusted OR = 0.80 (0.69–0.94), *P = *0.005; dominant: adjusted OR = 0.76 (0.64–0.92), *P = *0.003]. Further analyses of the combined genotypes of these six SNPs revealed a significant increase in PCa risk with increasing numbers of putative high-risk alleles (*P*_trend_ = 0.0005) (**Table 3**).

**Table 2:** Logistic regression analysis of associations between *mTOR* genotypes and prostate cancer risk in Eastern Chinese men.

Variables (HWE)^a^	Cases (N = 1004)	Controls (N = 1051)	*P* b	Crude OR (95% CI)	*P*	Adjusted OR (95% CI)^b^	*P* c
**rs2536 (HWE: *P* = 0.156)**							
TT	804 (80.1)	894 (85.1)	0.007	1.00		1.00	
CT	192 (19.1)	147 (14.0)		**1.45 (1.15–1.84)**	**0.002**	**1.45 (1.15–1.84)**	**0.002**
CC	8 (0.8)	10 (0.9)		0.89 (0.35–2.27)	0.806	0.88 (0.35–2.25)	0.795
Additive model				**1.34 (1.08–1.66)**	**0.008**	**1.34 (1.08–1.66)**	**0.008**
Dominant model			0.003	**1.42 (1.13–1.78)**	**0.003**	**1.42 (1.13–1.78)**	**0.003**
Recessive model			0.707	0.84 (0.33–2.13)	0.709	0.83 (0.33–2.12)	0.698
**rs1883965 (HWE: *P* = 0.904)**							
GG	843 (84.0)	890 (84.7)	0.874	1.00		1.00	
AG	153 (15.2)	154 (14.7)		1.05 (0.82–1.34)	0.700	1.06 (0.83–1.35)	0.640
AA	8 (0.8)	7 (0.7)		1.21 (0.44–3.34)	0.718	1.33 (0.48–3.70)	0.588
Additive model				1.06 (0.85–1.32)	0.622	1.08 (0.86–1.34)	0.522
Dominant model			0.655	1.06 (0.83–1.34)	0.655	1.07 (0.84–1.36)	0.574
Recessive model			0.728	1.20 (0.43–3.32)	0.728	1.32 (0.47–3.66)	0.600
**rs1034528 (HWE: *P* = 0.443)**							
GG	639 (63.7)	727 (69.2)	0.022	1.00		1.00	
CG	333 (33.2)	290 (27.6)		**1.31 (1.08–1.58)**	**0.006**	**1.31 (1.08–1.59)**	**0.005**
CC	32 (3.2)	34 (3.2)		1.07 (0.65–1.76)	0.787	1.09 (0.66–1.79)	0.739
Additive model				**1.20 (1.03–1.41)**	**0.023**	**1.21 (1.03–1.42)**	**0.019**
Dominant model			0.008	**1.28 (1.07–1.54)**	**0.008**	**1.29 (1.07–1.55)**	**0.007**
Recessive model			0.951	0.99 (0.60–1.61)	0.951	1.00 (0.61–1.64)	0.994
**rs17036508 (HWE: *P* = 0.085)**							
TT	749 (74.6)	820 (78.0)	0.135	1.00		1.00	
CT	237 (23.6)	210 (20.0)		1.24 (1.00–1.53)	0.049	1.23 (0.99–1.52)	0.055
CC	18 (1.8)	21 (2.0)		0.94 (0.50–1.78)	0.846	0.94 (0.49–1.77)	0.839
Additive model				1.15 (0.96–1.38)	0.128	1.15 (0.96–1.38)	0.139
Dominant model			0.068	1.21 (0.99–1.48)	0.069	1.20 (0.98–1.48)	0.076
Recessive model			0.733	0.90 (0.47-1.69)	0.734	0.89 (0.47–1.69)	0.731
**rs3806317 (HWE: *P* = 0.746)**							
AA	772 (76.9)	790 (75.2)	0.351	1.00		1.00	
AG	220 (21.9)	241 (22.9)		0.93 (0.76–1.15)	0.521	0.93 (0.76–1.15)	0.500
GG	12 (1.2)	20 (1.9)		0.61 (0.30–1.27)	0.186	0.61 (0.29–1.25)	0.174
Additive model				0.90 (0.75–1.08)	0.242	0.89 (0.74–1.07)	0.224
Dominant model			0.360	0.91 (0.74–1.11)	0.360	0.91 (0.74–1.11)	0.340
Recessive model			0.195	0.62 (0.30–1.28)	0.199	0.62 (0.30–1.27)	0.188
**rs2295080 (HWE: *P* = 0.334)**							
TT	653 (65.0)	617 (58.7)	0.012	1.00		1.00	
GT	311 (31.0)	382 (36.4)		**0.77 (0.64–0.93)**	**0.006**	**0.77 (0.64–0.93)**	**0.006**
GG	40 (4.0)	52 (5.0)		0.73 (0.47–1.11)	0.143	0.73 (0.48–1.12)	0.147
Additive model				**0.80 (0.69–0.93)**	**0.004**	**0.80 (0.69–0.94)**	**0.005**
Dominant model			0.003	**0.76 (0.64–0.91)**	**0.003**	**0.76 (0.64–0.92)**	**0.003**
Recessive model			0.291	0.80 (0.52–1.22)	0.292	0.8 (0.52–1.22)	0.300

OR, odds ratio; CI, confidence interval.

a Hard-Wenberg equilibrium test for controls.

b Two-sided Chi-square tests were used to calculate differences in the frequency distribution of genotypes between cases and controls.

c Adjusted for age, smoking, and BMI status in logistic regress models.

The results were in bold, if the 95% CI excluded 1 or *P* <0.05.

**Table 3:** Combined effects of risk genotypes of *mTOR* by dominant genetic models.

*mTOR* Variables	Cases	Controls	*P* a	Crude OR	*P*	Adjusted OR	*P* b
Genotypes	(N = 1004)	(N = 1051)		(95% CI)		(95% CI)^a^	
0–1	466 (46.4)	543 (51.7)	0.004	1.00		1.00	
2	295 (29.4)	322 (30.6)		1.07 (0.87–1.31)	0.523	1.07 (0.87–1.31)	0.516
3	162 (16.1)	129 (12.3)		**1.46 (1.13–1.90)**	**0.004**	**1.48 (1.14–1.93)**	**0.004**
4	79 (7.9)	53 (5.0)		**1.74 (1.20–2.51)**	**0.003**	**1.74 (1.20–2.52)**	**0.003**
5	2 (0.2)	4 (0.4)		0.58 (0.11–3.20)	0.534	0.52 (0.09–2.86)	0.450
						*P*_trend_ = 0.0005	
0–1	466 (46.4)	543 (51.7)	0.017	1.00		1.00	
≥2	538 (53.6)	508 (48.3)	?	**1.23 (1.04–1.47)**	**0.018**	**1.24 (1.04–1.47)**	**0.016**

a Chi-square test was used to calculate the genotype frequency distributions.

b Obtained under dominant models in logistic regression analyses with adjustment for age, smoking status and BMI.

The results were in bold, if the 95% CI excluded 1 or *P*<0.05.

### Stratification analysis of PCa risk associated with *mTOR* SNPs

In stratification analyses, as shown in **Tables 4** and **5**, the multivariate logistic regression analyses indicated, by assuming a dominant genetic model, that both *mTOR* rs2536 CT/CC and rs1034528 CG/CC genotypes were associated with an increased risk of PCa, particularly in subgroups of age≤69, BMI≤24 kg/m^2^, ever smokers, Gleason score≤7 (3+4), Gleason score≥7 (3+4), and stage III/IV disease, compared with their homozygous wild-type genotypes, respectively. The rs17036508 CT/CC genotypes were also associated with an increased risk of PCa among subgroups of BMI≤24 kg/m^2^, Gleason score≤7 (3+4), and stage III+IV diseases, compared with the TT homozygous variant genotype. In contrast, the rs2295080 GT/TT genotypes had a protective effect, particularly in subgroups of age>69, BMI, ever smokers, Gleason score≥7 (3+4), and stage I+II diseases, compared with the GG homozygous variant genotype. However, further homogeneity tests indicated that there was no difference in risk estimates between subgroups for most of the strata with a few exceptions including BMI level by rs2536 CT/CC genotypes [OR = 1.43 (1.13–1.80), *P* = 0.017] and by rs1034528 CC/CG genotypes [OR = 1.28 (1.07–1.55), *P* = 0.039]; disease stage by rs2536 CT/CC genotypes [OR = 1.47 (1.20–1.80), *P* = 0.004] and by rs17036508 CT/CC genotypes [OR = 1.24 (1.04–1.49), *P* = 0.024].

**Table 4:** Stratification analysis for associations between *mTOR* variants and prostate cancer risk by dominant genetic models in all subjects of Eastern Chinese men.

Variables	rs2536		Adjusted	*P* a	*P* ^hom^	rs1883965		Adjusted	*P* a	*P* ^hom^	rs1034528		Adjusted	*P* a	*P* ^hom^
	(cases/controls)		OR (95%CI)^a^			(cases/controls)		OR (95%CI)^a^			(cases/controls)		OR (95%CI)^a^		
	CT+CC	TT				AG+AA	GG				CG+CC	GG			
Age, yr (median)															
≤69	103/84	407/484	**1.42 (1.03–1.95)**	**0.031**	0.796	90/88	420/480	1.19 (0.86–1.65)	0.291	0.378	189/167	321/401	**1.40 (1.09–1.81)**	**0.010**	0.269
>69	97/73	397/410	1.38 (0.99–1.93)	0.057		71/73	423/410	0.94 (0.66–1.34)	0.716		176/157	318/326	1.16 (0.89–1.51)	0.287	
BMI, kg/m^2^															
≤24	159/85	595/552	**1.74 (1.30–2.32)**	**0.0002**	**0.017**	117/95	637/542	1.05 (0.79–1.42)	0.726	0.782	284/186	470/451	**1.47 (1.17–1.84)**	**0.001**	0.039
>24	41/72	209/342	0.93 (0.61–1.41)	0.736		44/66	206/348	1.13 (0.74–1.71)	0.578		81/138	169/276	0.95 (0.68–1.33)	0.776	
Smoking status															
Never	82/63	320/345	1.40 (0.98–2.02)	0.068	0.951	60/66	342/342	0.93 (0.63–1.36)	0.701	0.322	149/132	253/276	1.24 (0.93–1.66)	0.144	0.733
Ever	118/94	484/549	**1.42 (1.06–1.92)**	**0.021**		101/95	501/548	1.18 (0.87–1.60)	0.298		216/192	386/451	**1.32 (1.04–1.67)**	**0.023**	
Gleason score^b^															
≤7(3+4)	67/157	245/894	**1.58 (1.15–2.18)**	**0.005**	0.699	56/161	256/890	1.24 (0.89–1.74)	0.208	0.562	124/324	188/727	**1.51 (1.16–1.96)**	**0.002**	0.438
≥7(4+3)	121/157	480/894	**1.44 (1.11–1.87)**	**0.007**		97/161	504/890	1.07 (0.81–1.41)	0.635		220/324	381/727	**1.30 (1.05–1.60)**	**0.016**	
Stage of disease^c^															
I+II	68/157	368/894	1.07 (0.78–1.46)	0.672	**0.004**	79/161	357/890	1.25 (0.93–1.68)	0.142	0.339	147/324	289/727	1.16 (0.92–1.48)	0.214	0.078
III+IV	123/157	368/894	**1.91 (1.47–2.49)**	**<0.0001**		75/161	416/890	1.00 (0.75–1.35)	0.980		199/324	292/727	**1.53 (1.22–1.91)**	**0.0002**	

Variables	rs17036508		Adjusted	*P* a	*P* ^hom^	rs3806317		Adjusted	*P* a	*P* ^hom^	rs2295080		Adjusted	*P* a	*P* ^hom^
	(cases/controls)		OR (95%CI)^a^			(cases/controls)		OR (95%CI)^a^			(cases/controls)		OR (95%CI)^a^		
	CT+CC	TT				AG+GG	AA				GT+GG	TT			
Age, yr (median)															
≤69	129/120	381/448	1.23 (0.92–1.63)	0.161	0.642	113/140	397/428	0.86 (0.65–1.14)	0.296	0.674	195/247	315/321	0.79 (0.62–1.01)	0.055	0.599
>69	126/111	368/372	1.16 (0.87–1.56)	0.320		119/121	375/362	0.94 (0.70–1.25)	0.659		156/187	338/296	**0.74 (0.57–0.96)**	**0.024**	
BMI, kg/m^2^															
≤24	200/139	554/498	**1.29 (1.01–1.66)**	**0.042**	0.242	172/164	582/473	0.85 (0.67–1.09)	0.206	0.398	271/265	483/372	**0.79 (0.64–0.98)**	**0.035**	0.476
>24	55/92	195/322	0.98 (0.67–1.43)	0.918		60/97	190/317	1.03 (0.71–1.48)	0.896		80/169	170/245	**0.68 (0.49–0.94)**	**0.021**	
Smoking status															
Never	108/96	294/312	1.19 (0.86–1.63)	0.288	0.932	98/103	304/305	0.95 (0.69–1.31)	0.747	0.695	152/173	250/235	0.83 (0.62–1.10)	0.187	0.473
Ever	147/135	455/508	1.21 (0.93–1.58)	0.165		134/158	468/485	0.86 (0.66–1.12)	0.262		199/261	403/382	**0.72 (0.57–0.91)**	**0.006**	
Gleason score^b^															
≤7(3+4)	90/231	222/820	**1.44 (1.08–1.92)**	**0.013**	0.253	75/261	237/790	0.95 (0.71–1.27)	0.720	0.480	121/434	191/617	0.91 (0.70–1.18)	0.465	0.289
≥7(4+3)	148/231	453/820	1.16 (0.92–1.47)	0.219		130/261	471/790	0.84 (0.66–1.06)	0.140		208/434	393/617	**0.75 (0.61–0.93)**	**0.008**	
Stage of disease^c^															
I+II	95/231	341/820	1.00 (0.76–1.31)	0.970	**0.024**	101/261	335/790	0.90 (0.69–1.17)	0.439	0.628	142/434	294/617	**0.69 (0.55–0.88)**	**0.002**	0.092
III+ IV	146/231	345/820	**1.50 (1.18–1.91)**	**0.001**		106/261	385/790	0.83 (0.64–1.08)	0.158		191/434	300/617	0.90 (0.73–1.13)	0.364	

BMI, body mass index.

a Obtained under dominant models in logistic regression analyses with adjustment for age, smoking status and BMI.

b,c According to the current WHO recommendations.

*P* ^hom^ *P* value for homogeneiy test.

The results were in bold, if *P*<0.05.

**Table 5:** Stratification analysis for associations between combined risk genotypes of *mTOR* variants and prostate cancer risk.

Variables	Combined effect of risk genotypes (cases/controls)		Crude OR(95%CI)	*P*	Adjusted OR(95%CI)^a^	*P* a	*P* b	Interaction
	0–1 at-risk genotype	2-6 at-risk genotype						*P* c
Age, yr								
≤69 (median)	236/301	274/267	1.31 (1.03–1.66)	0.028	**1.29 (1.02–1.65)**	**0.036**	0.473	0.872
>69 (median)	230/242	264/241	1.15 (0.90–1.48)	0.268	1.15 (0.90–1.48)	0.271		
BMI, kg/m^2^								
≤24	348/336	406/301	1.30 (1.05–1.61)	0.014	**1.31 (1.06–1.61)**	**0.013**	0.431	0.431
>24	118/207	132/207	1.12 (0.82–1.53)	0.484	1.11 (0.81–1.52)	0.527		
Smoking status								
Never	185/201	217/207	1.14 (0.86–1.50)	0.356	1.15 (0.87–1.52)	0.322	0.470	0.470
Ever	281/342	321/301	1.30 (1.04–1.62)	0.022	**1.29 (1.03–1.61)**	**0.027**		
Gleason score								
≤7(3+4)	133/543	179/508	1.44 (1.12–1.86)	0.005	**1.46 (1.13–1.89)**	**0.004**	0.258	
≥7(4+3)	284/543	317/508	1.19 (0.98–1.46)	0.085	1.19 (0.98–1.46)	0.084		
Stage of disease								
I+II	208/543	228/508	1.17 (0.94–1.47)	0.165	1.18 (0.95–1.48)	0.140	0.400	
III+ IV	218/543	273/508	1.34 (1.08–1.66)	0.008	**1.34 (1.08–1.66)**	**0.008**		

a Obtained in logistic dominant models with adjustment for age, smoking status and BMI.

b *P* for homogeneity test using the χ2-based Q test.

c Test for multiplicative interaction obtained from logistic regression models with adjustment for age, smoking status and BMI.

CI, confidence interval; BMI, body mass index.

The results were in bold, if *P*<0.05.

### Haplotype analysis of the *mTOR* SNPs

Based on the genotyping results to infer possible haplotypes, we used the four SNPs (rs2536 T>C, rs1034528 G>C, rs17036508 T>C, and rs2295080 T>G) that were statistically significantly associated with PCa risk in the single locus analysis (**Table 6**). When the common “TGTT” haplotype was used as the reference, the “CCCG” haplotype was associated with an evidently increased PCa risk [adjusted OR = 1.31 (1.03–1.66), *P = *0.026] However, the “TGTG” and “TGCG” haplotypes were associated with an evidently decreased but not increased PCa risk, with the adjusted OR of 0.39 (0.27–0.56), *P* <0.0001 and 0.63 (0.43–0.91), *P = *0.014, respectively. The findings of haplotypes “CCTG” and “CCCT” may not be reliable due to their relatively small numbers of observations.

**Table 6:** The frequency of common inferred haplotrypes of the *mTOR* gene based on the observed genotypes.

rs2536	rs1034528	rs17036508	rs2295080	Case (N = 2014)	Control (N = 2134)	Adjusted OR (95% CI)	*P* a
T	G	T	T	1515	1572	1.00	
T	G	T	G	41	109	0.39 (0.27–0.56)	<0.000
T	G	C	T	12	7	1.77 (0.70–4.51)	0.231
T	G	C	G	46	76	0.63 (0.43–0.91)	0.014
T	C	T	T	55	40	1.44 (0.95–2.17)	0.087
T	C	T	G	127	145	0.92 (0.71–1.17)	0.481
T	C	C	T	3	2	1.48 (0.24–8.90)	0.667
T	C	C	G	6	14	0.43 (0.16–1.11)	0.081
C	G	T	T	0	2	-	-
C	G	T	G	0	2	-	-
C	G	C	T	0	1	-	-
C	G	C	G	0	2	-	-
C	C	T	T	1	2	0.55 (0.05–6.03)	0.622
C	C	T	G	1	9	0.11 (0.01–0.89)	0.039
C	C	C	T	36	15	2.47 (1.34–4.52)	0.004
C	C	C	G	171	136	1.31 (1.03–1.66)	0.026


a Obtained under dominant models in logistic regression analyses with adjustment for age, smoking status and BMI.

The results were in bold, if the 95% CI excluded 1 or *P*<0.05.

### Association of high-order interactions with PCa

To further explore high-order interactions, we performed the MDR analyses by including the genotypes of four significant *mTOR* SNPs (i.e., rs2536 CT/CC, rs1034528 CG/CC, rs17036508 CT/CC, and rs2295080 GT/GG vs. their wild-type homozygotes, respectively) and three risk factors (i.e., age at diagnosis, smoking status, and BMI). The results showed that BMI was the best one-factor model with the highest CVC (100%) and the lowest prediction error (42.7%) among all seven factors. Likewise, the interaction between BMI, rs17036508 T>C, and rs2536 T>C was the best three-factor model involving both environmental and genetic factors with the highest CVC (100%) and the lowest prediction error of 41.9% (**Table 7**).

**Table 7:** MDR analysis for the risk of prostate cancer prediction in an Eastern Chinese population.

est interaction models	Cross-validation	Average prediction error	*P*-value^a^
BMI	100/100	42.7%	<0.0001
BMI, rs2295080	100/100	42.7%	<0.0001
**BMI, rs17036508, rs2536**	**100/100**	**41.9%**	**<0.0001**
BMI, rs17036508, rs2536, rs2295080	99/100	41.5%	<0.0001
BMI, rs17036508, rs2536, rs2295080, smoking status	95/100	41.1%	<0.0001
BMI, rs17036508, rs2536, rs2295080, smoking status, age	90/100	40.7%	<0.0001
BMI, rs17036508, rs2536, rs2295080, rs1034528, smoking status, age	99/100	40.2%	<0.0001

MDR, multifactor dimensionality reduction.

The best model with maximum cross-validation consistency and minimum prediction error rate was in bold.

a *P*-value for 1000-fold permutation test.

Finally, the FPRP values at different prior probability levels for all significant findings are summarized in **Table 8**. When the assumption of prior probability was 0.01, the association with rs2536 (CT/CC vs. TT) was noteworthy in subgroups of≤24 kg/m^2^ BMI and stage III+IV (FPRP = 0.112 and 0.055, respectively), and the similar results can be observed in the association with rs1034528 (CG/CC vs. GG) in subgroups of≤24 kg/m^2^ BMI and stage III+IV (FPRP = 0.132 and 0.043, respectively) as well as the association with subgroup of stage III+IV (FPRP = 0.165) by rs17036508 (CT/CC vs. TT). In contrast, some greater FPRP values for the other significant associations between *mTOR* variants and prostate cancer risk suggested some possible bias in the findings, which need further validation in larger studies.

**Table 8:** False-positive report probability values for associations between the risk of cancer and the frequency of genotypes of *mTOR* variants.

mTOR SNP genotype	Crude OR (95%CI)	*P* a	Statistical power^b^	Prior probability				
				0.25	0.1	0.01	0.001	0.0001
All patients								
rs2536, CT vs TT	1.45 (1.15–1.84)	0.0018	0.614	**0.009**	**0.026**	0.225	0.746	0.967
rs1034528, CG vs GG	1.31 (1.08–1.58)	0.0058	0.93	**0.018**	**0.053**	0.382	0.862	0.984
rs2295080, GT vs TT	1.24 (1.03–1.49)	0.0203	0.982	**0.058**	**0.157**	0.672	0.954	0.995
rs2295080, GG vs TT	0.09 (0.02–0.30)	0.0001	0.007	**0.044**	**0.12**	0.601	0.938	0.993
rs2295080, GG vs GT/TT	0.09 (0.03–0.28)	0.0001	0.008	**0.036**	**0.101**	0.552	0.926	0.992
rs2536, CT/CC vs TT								
All patients	1.42 (1.13–1.78)	0.0029	0.699	**0.012**	**0.036**	0.291	0.806	0.976
Age≤69 yrs	1.46 (1.06–2.00)	0.0192	0.573	**0.091**	0.232	0.768	0.971	0.997
BMI≤24 kg/m^2^	1.74 (1.30–231)	0.0002	0.157	**0.004**	**0.011**	**0.112**	0.559	0.927
Ever smoking	1.42 (1.06–1.92)	0.0194	0.642	**0.083**	0.214	0.749	0.968	0.997
Gleason score≤7(3+4)	1.56 (1.13–2.14)	0.0062	0.406	**0.044**	**0.121**	0.602	0.938	0.993
Gleason score≥7(4+3)	1.44 (1.11–1.87)	0.0066	0.634	**0.030**	**0.086**	0.507	0.912	0.990
Stage III+ IV	1.90 (1.46–2.48)	0.0001	0.17	**0.002**	**0.005**	**0.055**	0.371	0.855
rs1034528, CG/CC vs GG								
All patients	1.28 (1.07–1.54)	0.0080	0.958	**0.024**	**0.07**	0.452	0.893	0.988
Age≤69 yrs	1.41 (1.10–1.82)	0.0076	0.681	**0.032**	**0.091**	0.525	0.918	0.991
BMI≤24 kg/m^2^	1.47 (1.17–1.84)	0.0009	0.586	**0.005**	**0.014**	**0.132**	0.605	0.939
Ever smoking	1.31 (1.04–1.67)	0.0237	0.870	**0.076**	**0.197**	0.730	0.965	0.996
Gleason score≤7(3+4)	1.48 (1.14–1.92)	0.0032	0.539	**0.017**	**0.051**	0.370	0.856	0.983
Gleason score≥7(4+3)	1.30 (1.05–1.60)	0.0162	0.917	**0.050**	**0.137**	0.636	0.946	0.994
Stage III+ IV	1.53 (1.22–1.91)	0.0002	0.443	**0.001**	**0.004**	**0.043**	0.311	0.819
rs17036508, CT/CC vs TT								
BMI≤24 kg/m^2^	1.29 (1.01–1.66)	0.0417	0.892	**0.123**	0.296	0.822	0.979	0.998
Gleason score≤7(3+4)	1.44 (1.08–1.91)	0.0121	0.615	**0.056**	**0.151**	0.661	0.952	0.995
Stage III+ IV	1.50 (1.18–1.91)	0.0010	0.499	**0.006**	**0.018**	**0.165**	0.667	0.952
rs2295080, GT/GG vs TT								
Age≤69 yrs	1.30 (1.01–1.66)	0.0380	0.885	**0.114**	0.279	0.810	0.977	0.998
Gleason score≤7(3+4)	1.34 (1.03–1.73)	0.0274	0.815	**0.092**	0.232	0.769	0.971	0.997
Stage III+ IV	1.35 (1.08–1.68)	0.0074	0.885	**0.024**	**0.070**	0.453	0.893	0.988
Combined effect								
4 variable genotypes	1.23 (1.04–1.47)	0.017	0.988	**0.052**	**0.141**	0.643	0.948	0.995
mTOR haplotypes (rs2536-rs1034528-rs17036508-rs2295080)								
T-G-T-G	0.39 (0.27–0.56)	<0.0001	0.088	**0.003**	**0.010**	**0.101**	0.531	0.919
T-G-C-G	0.63 (0.43–0.91)	0.0137	0.378	**0.098**	0.246	0.782	0.973	0.997
C-C-C-G	1.31 (1.03–1.65)	0.0269	0.895	**0.083**	0.213	0.748	0.968	0.997

OR, odds ratio; CI, confidence interval; BMI, body mass index.

a Chi-square test was used to calculate the genotype frequency distributions.

b Statistical power was calculated using the number of observations in the subgroup and the OR and *P* values in this table.

The results in false-positive report probability analysis were in bold, if the prior probability < 0.2.

## Discussion

In this large, ethnic specific single institutional case-control study, we investigated the associations between six potentially functional SNPs of the *mTOR* gene and PCa risk, and we found that the rs2536 C, rs1034528 C, and rs2295080 G variant genotypes were associated with PCa risk, and the effects were more evident in subgroups of age≤69, BMI≤24 kg/m^2^, and ever-smokers. Additionally, the variant genotypes were more common in patients with high-grade diseases (stage III+IV), indicating their likely involvement in the development and progression of PCa. To the best of our knowledge, this is the first post-GWAS study that focused on the associations of these six potentially functional *mTOR* SNPs with PCa risk.

The *mTOR* gene, located on chromosome 1p36.2, encodes a protein kinase product of 289 kDa and has emerged as a critical cell growth effector by controlling mRNA translation, ribosome biogenesis, autophagy, and metabolism [21]–[23]. Studies have shown that there are some important domains ranging from the *N*- to the *C*-terminus of mTOR. For example, the *N*-terminus of mTOR contains two tandem repeated HEAT motifs that can mediate interactions between proteins, the FAT domain that can facilitate focal adhesion to the targeting domain, and the FRB domain that is regarded as one high-affinity binding site for the inhibitory complex FKBP12-rapamycin, whereas there are two regulatory domains that are located at the *C*-terminus of the protein including PtdIns 3-kinase related catalytic domain and FATC domain [13]. To date, there are 2651 SNPs that have been observed scattered across the whole *mTOR* gene. Given the critical role of mTOR in the PNET/AKT/mTOR signaling pathway, it is biologically plausible that functional SNPs affecting the pivotal domains described above may contribute to cancer susceptibility. However, in addition to the published GWAS studies, only a few reported post-GWAS studies have investigated the associations between functional SNPs of the *mTOR* gene and risk of PCa. In a Chinese study with 666 PCa and 708 cancer-free controls, Chen et al. [24] indicated that *mTOR* rs2295080 GT/GG genotypes had a protective effect on PCa risk, compared with the TT genotype, which was recently shown by Xu et al. in gastric cancer [25] and by Cao et al. in renal cell cancer [26]. These findings are consistent with those of the present study with a larger sample size. Additionally, Hildebrandt et al. found that individuals carrying the rs2295080 GG genotype had reversed clinical outcomes in Caucasian esophageal cancer patients treated with chemotherapy, compared with the TT homozygous wild-type genotype [27]. All studies that focused on Chinese populations indicated an association between rs2295080 GT/GG and cancer risk, suggesting an possibly ethnic-specific association. Nevertheless, the associations between *mTOR* rs2536 C variant or genotypes and cancer risk in Chinese populations were various in the literatures; for example, the *mTOR* rs2536 CT heterozygous genotype was found to be associated with decreased risk of Chinese childhood acute lymphoblastic leukemia [28]; however, this association was not observed in other tumor types, such as gastric cancer [29], prostate cancer [24], and esophageal squamous cell carcinoma [30]. On the contrary, in the present study, we found that the *mTOR* rs2536 CT/CC genotypes were associated with an increased PCa risk under a dominant genetic model, different from the findings of another previously published PCa study (666 cases and 708 controls), in which a null association was reported [24]. We speculated that the disagreement might be due to the different sample size or different inclusion criteria for the participaion, which needs large and better designed studies to confirm.

Studies have shown that the rs2295080 T allele could enhance the transcription activity of *mTOR* in HEK293, 786-O, HeLa, and GES-1 cell line *in vitro* [25], [26]. Likewise, individuals carrying the TT genotype had higher levels of *mTOR* expression as well [25], [26]. These suggest that the rs2295080 T allele could increase the affinity of special transcription factors to this region of the *mTOR* promoter and subsequently contribute to the increased *mTOR* activity in humans. Theoretically, miRNAs can bind to the 3′ UTR of target genes and inhibit gene expression translationally and/or by destabilizing the target mRNA. Based on a bioinformatics web server (http://snpinfo.niehs.nih.gov/cgi-bin/snpinfo), the SNP rs2536 T>C was predicted to bind to miRNA-576 at the T variant allele or bind to miRNA-767 at the C allele. Therefore, we speculated that the expression of mTOR depended on the proportions of these two miRNAs or the affinity between miRNA and SNP rs2536 T>C, which has a growth advantage of immortalized cells and induces neoplastic transformation. It was indicated that disease-associated functional intronic variants may alter mRNA levels of the genes by affecting the transcriptional efficiency, RNA elongation, or splicing [31]–[33]. On the other hand, the SNP rs1034528 G>C located in the first intron region of the *mTOR* gene was also found to be associated with risk of PCa; however, both rs1034528 G and C alleles were predicted to bind to different transcription factors, respectively, in this region. Therefore, the exact mechanisms of the rs1034528 G>C underlying the observed PCa risk need additional functional studies.

There is evidence in the literature that each SNP may have a weak effect but the combination of multi-SNPs may present much stronger effects than any of the SNPs. This is particularly true in the present study, in which the haplotype and combined analyses confirmed the multi-SNPs effects in PCa. In the logistic regression model, a locus dose-response was found for the increased PCa risk with the increasing number of adverse genotypes of all studied SNPs. Additionally, we noticed the combined effects was more pronounced among subgroups of age≤69 and BMI≤24 kg/m^2^. These findings agreed with the hypothesis that genetic susceptibility contributes to the risk of developing cancer in those who had an early age onset and minor exposures. Although the interaction between smoking and *mTOR* SNPs was not observed in the present study, we did find an obvious effect of the combined unfavorable genotypes on PCa risk, particularly among subgroups of ever smoker, suggesting that the effect of the tobacco smoke-related carcinogens may also depend on genetic factors.

In the present study, the number of positive findings from the stratified analyses was obviously decreased in the FPRP assessment. There are several possible explanations for the false positive findings. Firstly, some findings in the stratified analyses may be a chance finding due to the limited sample size in the subgroups. Secondly, some missing information and potential confounding factors might result in the false positive associations. Therefore, all positive results should be explained with caution. Extensive evidence from previous epidemiology studies has indicated that several genetic variant and environmental factors are involved in the initiation and development of cancer [34]–[37]. We also found the similar interactions by using logistic regression and MDR approaches (**Table S1 in File S1**). In the MDR analysis, BMI was found to be the most noteworthy factor in one-factor model; however, the exact mechanisms for the association between BMI and PCa risk have not been established. Possible hypotheses include the effect of hormones, PSA, and adipose-related proteins [38]. In the present study, we found some evidence of the interactions between environmental factor (BMI) and genetic factors (rs17036508 T>C and rs2536 T>C), as shown in the best three-factor model, we speculated that those variations might alter the expression of *mTOR* and the subsequent synthesis of adipose-related proteins, but this finding needs to be validated in larger studies.

In summary, the present study investigated the associations between six selected potentially functional *mTOR* SNPs and PCa risk with a relative large sample size. However, several methodological issues and limitations of the present study should be discussed. Firstly, some participants might be misclassified due to the lack of PSA serum information; for example, some silent tumors (stage A1, usually asymptomatic) may have been included as normal controls, which could subsequently bias the results to the null. Secondly, although hormonal, occupational, dietary, inflammation and other factors have been suggested as etiological factors of PCa, we did not adequately documented these covariables for adjustment. Thirdly, only six potentially functional SNPs of *mTOR* were investigated in the present study, which did not cover all variants in the *mTOR* gene. Therefore, additional larger and well-designed studies are warranted to confirm our findings.

## Supporting Information

File S1 File includes: Supplementary Table S1 for Stratification analysis of significant SNPs by age, smoking status, and BMI; S1-1 Stratification analysis of significant SNPs by age; S1-2 Stratification analysis of significant SNPs by smoking status; and S1-3 Stratification analysis of significant SNPs by BMI.
(DOCX)
