# Correction: Polymorphisms in the *mTOR* Gene and Risk of Sporadic Prostate Cancer in an Eastern Chinese Population

**DOI:** 10.1371/annotation/bc36c048-60e4-48a2-908d-6253e93df062

**Published:** 2014-01-06

**Authors:** Qiaoxin Li, Chengyuan Gu, Yao Zhu, Mengyun Wang, Yajun Yang, Jiucun Wang, Li Jin, Mei-Ling Zhu, Ting-Yan Shi, Jing He, Xiaoyan Zhou, Ding-wei Ye, Qingyi Wei

Affiliations numbers 1 and 3 for multiple authors are incorrect. The correct affiliation number 1 is: Cancer Institute, Fudan University Cancer Center, Shanghai, China. The correct affiliation number 3 is: Department of Urology, Fudan University Shanghai Cancer Center, Shanghai, China. 

