# Supplementary material for: Polymorphisms in the mTOR Gene and Risk of Sporadic Prostate Cancer in an Eastern Chinese Population
Source: PLoS One. 2013 Aug 5;8(8):e71968. doi: 10.1371/journal.pone.0071968 (PMC3734314; doi:10.1371/journal.pone.0071968)
Supplement: File S1 — File includes: Supplementary Table S1 for Stratification analysis of significant SNPs by age, smoking status, and BMI; S1-1 Stratification analysis of significant SNPs by age; S1-2 Stratification analysis of significant SNPs by smoking status; and S1-3 Stratification analysis of significant SNPs by BMI. (DOCX) [file pone.0071968.s001.docx]

| **Supplementary Table S1 for Stratification analysis**^#^ **of significant SNPs by age, smoking status, and BMI**  **S1-1** Stratification analysis of significant SNPs by age | | | | | |
| --- | --- | --- | --- | --- | --- |
| **variation** | **Age** | **Cases** | **Controls** | **OR (95% CI)^*^** | **Interaction** |
|  | **yr (median)** | **(n=1015)** | **(n=1044)** |  | ***P^a^*** |
| rs2536 | | | | | 0.796 |
| TT | ≤ 69 | 407 | 484 | 1.00 |  |
| TT | >69 | 397 | 410 | 1.12 (0.93-1.36) |  |
| CT/CC | ≤69 | 103 | 84 | 1.44 (1.05-1.98) |  |
| CT/CC | >69 | 97 | 73 | 1.56 (1.12-2.18) |  |
| rs1883965 | | | | | 0.378 |
| GG | ≤69 | 420 | 480 | 1.00 |  |
| GG | >69 | 423 | 410 | 1.16 (0.96-1.40) |  |
| AG/AA | ≤69 | 90 | 88 | 1.19 (0.86-1.64) |  |
| AG/AA | >69 | 71 | 73 | 1.09 (0.77-1.55) |  |
| rs1034528 | | | | | 0.269 |
| GG | ≤69 | 321 | 401 | 1.00 |  |
| GG | >69 | 318 | 326 | 1.19 (0.96-1.48) |  |
| CG/CC | ≤69 | 189 | 167 | 1.42 (1.10-1.83) |  |
| CG/CC | >69 | 176 | 157 | 1.38 (1.06-1.80) |  |
| rs17036508 | | | | | 0.642 |
| TT | ≤69 | 381 | 448 | 1.00 |  |
| TT | >69 | 368 | 372 | 1.13 (0.93-1.38) |  |
| CT/CC | ≤69 | 129 | 120 | 1.25 (0.94-1.66) |  |
| CT/CC | >69 | 126 | 111 | 1.32 (0.98-1.76) |  |
| rs3806317 | | | | | 0.674 |
| AA | ≤69 | 397 | 428 | 1.00 |  |
| AA | >69 | 375 | 362 | 1.09 (0.89-1.34) |  |
| AG/GG | ≤69 | 113 | 140 | 0.87 (0.66-1.16) |  |
| AG/GG | >69 | 119 | 121 | 1.04 (0.78-1.38) |  |
| rs2295080 0.599 | | | | | |
| TT | ≤69 | 315 | 321 | 1.00 |  |
| TT | >69 | 338 | 296 | 1.13 (0.91-1.41) |  |
| GT/GG | ≤69 | 195 | 247 | 0.80 (0.63-1.02) |  |
| GT/GG | >69 | 156 | 187 | 0.83 (0.64-1.08) |  |

| **S1-2** Stratification analysis of significant SNPs by smoking status | | | | | |
| --- | --- | --- | --- | --- | --- |
| **variation** | **Smoking status** | **Cases** | **Controls** | **OR (95% CI)^*^** | **Interaction** |
|  |  | **(n=1015)** | **(n=1044)** |  | ***P^a^*** |
| rs2536 | | | | | 0.951 |
| TT | Never | 320 | 345 | 1.00 |  |
| TT | Ever | 484 | 549 | 0.97 (0.80-1.18) |  |
| CT/CC | Never | 82 | 63 | 1.41 (0.98-2.02) |  |
| CT/CC | Ever | 118 | 94 | 1.38 (1.01-1.88) |  |
| rs1883965 | | | | | 0.322 |
| GG | Never | 342 | 342 | 1.00 |  |
| GG | Ever | 501 | 548 | 0.93 (0.77-1.13) |  |
| AG/AA | Never | 60 | 66 | 0.93 (0.64-1.36) |  |
| AG/AA | Ever | 101 | 95 | 1.09 (0.79-1.50) |  |
| rs1034528 | | | | | 0.734 |
| GG | Never | 253 | 276 | 1.00 |  |
| GG | Ever | 386 | 451 | 0.95 (0.76-1.19) |  |
| CG/CC | Never | 149 | 132 | 1.24 (0.93-1.66) |  |
| CG/CC | Ever | 216 | 192 | 1.26 (0.97-1.63) |  |
| rs17036508 | | | | | 0.932 |
| TT | Never | 294 | 312 | 1.00 |  |
| TT | Ever | 455 | 508 | 0.97 (0.79-1.19) |  |
| CT/CC | Never | 108 | 96 | 1.19 (0.86-1.63) |  |
| CT/CC | Ever | 147 | 135 | 0.17 (0.88-1.56) |  |
| rs3806317 | | | | | 0.696 |
| AA | Never | 304 | 305 | 1.00 |  |
| AA | Ever | 468 | 485 | 0.99 (0.81-1.22) |  |
| AG/GG | Never | 98 | 103 | 0.96 (0.70-1.33) |  |
| AG/GG | Ever | 134 | 158 | 0.86 (0.65-1.14) |  |
| rs2295080 0.473 | | | | | |
| TT | Never | 250 | 235 | 1.00 |  |
| TT | Ever | 403 | 382 | 1.01 (0.81-1.27) |  |
| GT/GG | Never | 152 | 173 | 0.83 (0.63-1.10) |  |
| GT/GG | Ever | 199 | 261 | 0.73 (0.56-0.94) |  |

| **S1-3** Stratification analysis of significant SNPs by BMI | | | | | |
| --- | --- | --- | --- | --- | --- |
| **variation** | **BMI, kg/m^2^** | **Cases** | **Controls** | **OR (95% CI)*** | **Interaction** |
|  |  | **(n=1015)** | **(n=1044)** |  | ***P^a^*** |
| rs2536 | | | | | **0.017** |
| TT | ≤24 | 595 | 552 | 1.00 |  |
| TT | >24 | 209 | 342 | 0.57 (0.46-0.70) |  |
| CT/CC | ≤24 | 159 | 85 | 1.74 (1.30-2.31) |  |
| CT/CC | >24 | 41 | 72 | 0.53 (0.36-0.80) |  |
| rs1883965 | | | | | 0.783 |
| GG | ≤24 | 637 | 542 | 1.00 |  |
| GG | >24 | 206 | 348 | 0.51 (0.41-0.63) |  |
| AG/AA | ≤24 | 117 | 95 | 1.06 (0.79-1.42) |  |
| AG/AA | >24 | 44 | 66 | 0.57 (0.39-0.85) |  |
| rs1034528 | | | | | **0.039** |
| GG | ≤24 | 470 | 451 | 1.00 |  |
| GG | >24 | 169 | 276 | 0.59 (0.47-0.75) |  |
| CG/CC | ≤24 | 284 | 186 | 1.47 (1.17-1.84) |  |
| CG/CC | >24 | 81 | 138 | 0.57 (0.42-0.77) |  |
| rs17036508 | | | | | 0.242 |
| TT | ≤24 | 554 | 498 | 1.00 |  |
| TT | >24 | 195 | 322 | 0.55 (0.44-0.68) |  |
| CT/CC | ≤24 | 200 | 139 | 1.29 (1.01-1.66) |  |
| CT/CC | >24 | 55 | 92 | 0.54 (0.38-0.77) |  |
| rs3806317 | | | | | 0.398 |
| AA | ≤24 | 582 | 473 | 1.00 |  |
| AA | >24 | 190 | 317 | 0.49 (0.40-0.61) |  |
| AG/GG | ≤24 | 172 | 164 | 0.85 (0.67-1.09) |  |
| AG/GG | >24 | 60 | 97 | 0.50 (0.36-0.71) |  |
| rs2295080 0.476 | | | | | |
| TT | ≤24 | 483 | 372 | 1.00 |  |
| TT | >24 | 170 | 245 | 0.54 (0.43-0.69) |  |
| GT/GG | ≤24 | 271 | 265 | 0.79 (0.64-0.98) |  |
| GT/GG | >24 | 80 | 169 | 0.37 (0.27-0.50) |  |
| *Data were calculated by unconditional logistic regression with adjustment for age, smoking status and BMI.  ^a^: *p* value for Factor 1 × Factor 2 presents a test for the interaction between Factor 1 and Factor 2. The results were in bold, if *P*<0.05. | | | | | |
